# Farm Injury Deaths and Workers' Compensation Claims in Australia and Their Economic Costs

**DOI:** 10.1111/ajr.70087

**Published:** 2025-09-10

**Authors:** Carlos Mesa‐Castrillon, Kerri‐Lynn Peachey, Tony Lower

**Affiliations:** ^1^ AgHealth Australia, School of Rural Health, Faculty of Medicine and Health The University of Sydney Sydney New South Wales Australia

**Keywords:** aging, agriculture, cost analysis, farm, injury, workers' compensation

## Abstract

**Objective:**

To describe the pattern and estimated direct economic burdens associated with unintentional deaths and injuries on Australian farms over the past 11 years (2013–2023).

**Design:**

Descriptive retrospective epidemiological study of National Coronial Information System (NCIS) data for persons fatally injured on a farm and workers' compensation injuries data from the National Data Set.

**Setting:**

Australia.

**Participants:**

All agricultural cases involving fatal injury events and those being injured accessing workers compensation.

**Main Outcome Measures:**

Nature of fatal and injury events, with estimates on the economic costs associated with deaths and workers' compensation injury claims costs.

**Results:**

There were 748 farm fatalities, with 544 (73%) being work‐related. From these, 513 (94%) of the cases occurred in males, with almost half (48%) in farmers aged 60 years or older. The leading agents for fatalities were tractors (*n* = 118), quad‐bikes (*n* = 117) and farm utilities (*n* = 52). Costs for all fatalities (work and non‐work), approached $1.8 billion in the 2013‐2023 period (~$164 million per year). Work‐related fatalities accounted for $1.24 billion of this total, with an annual cost of approximately $112 million. There were around 5000 workers' compensation injury claims processed per year during 2013–2021, costing over $1.5 billion (~$190 million per year).

**Conclusion:**

The costs for all on‐farm injury deaths and workers' compensation injury claims conjointly during the period of 2013–2023, includes a conservative annual estimate of $355 million per year. Of this sum, approximately $300 million involved work‐related incidents. Although there is a modest progression in reducing farm deaths and injuries, targeted and evidence‐based approaches are required to stimulate improvements in these preventable incidents.

**Summary**

- What is already known on this subject?
  ○ Agriculture is a high‐risk industry, with males and older farmers dominating the fatal injury cases.
  ○ Key agents of concern continue to be farm vehicles and machinery.
- What this paper adds
  ○ This is the first assessment of all on‐farm “unintentional” injury related deaths (work and non‐work), in conjunction with a compilation of workers' compensation injury data in Australia.
  ○ Provides detailed direct estimates of costs for all on‐farm injury deaths and workers' compensation injury claims.
  ○ Information on agents of fatal injury by work status, the estimated economic cost of farm‐related fatalities, plus information on workers' compensation injury claims such as agency, nature, mechanism and bodily location of injuries.

## 1 Introduction

Agriculture is a cornerstone of Australian rural communities, employment and economies, with the industry seeking to exceed $100 billion in revenue per annum by 2030 [1]. Indeed, this target was approached in 2021–2022 and 2022–2023, but declined slightly in 2023–2024 [2]. Considerable work is also occurring to develop and enact sustainability frameworks, with several commodity sectors having established or being in the process of developing such plans [3, 4]. There is also a proposal for a whole of agriculture plan involving multiple commodity sectors—Australian Agricultural Sustainability Framework [5]. Within all of these strategies, the health, safety and wellbeing of people involved in the industry are core targets for attention.

Data for the period from 2001 to 2020 in Australia indicate an average of 79 on‐farm deaths per year, with little improvement since 2007 [6]. In comparison to other industry sectors, data for 2022–2023 report that agriculture (11.7 deaths/100 000 workers) is the second most dangerous sector, ranking only behind road transport (13.0). Other known high‐risk industries such as construction (2.8) and mining (3.6) have considerably lower fatality rates, which have reduced from similar levels to that currently reported for agriculture [7]. Relatedly, serious injury claims for 2022–2023 are highest in the agriculture, fisheries and forestry industry subdivision (noting agriculture is not separated in this calculation) at 20.9 claims/million hours worked. This compares to figures for construction (17.2), transport (17.1) and mining (15.2) [8].

Current repositories reporting work‐related fatal cases within Australian industries are maintained by Safe Work Australia [SWA]—(Traumatic Injuries Fatalities database—TIF) [9]. Data in the TIF are compiled according to SWA's scope, which includes the collation of workers' compensation data to develop national policy to improve work health and safety across Australia. There can be differences between the records SWA designates as work‐related and determinations made by the National Coronial Information System (NCIS) or Work Health Authorities (WHA) in each state/territory (jurisdiction). Deaths that are not work‐related, involve self‐harm or natural causes, are excluded from the TIF.

Many farmers continue to work well past what would conventionally be considered “retirement age” in other professions [10]. While they may not be drawing a wage, they continue their work roles. The jurisdictional WHA use their discretion in defining whether cases that involve this age cohort are formally defined as work‐related or not. Furthermore, there are regular cases (both work and non‐work) that involve people of all ages living on or visiting farms. This is important as the “farm” is the place of usual residence for a significant number of people involved in agriculture, with a commensurate increase in risk exposure to common agents of injury (tractors, vehicles, animals, etc.). Consequently, the inclusion of all on‐farm cases (work and non‐work) is necessary to provide a full review of the current status of deaths on Australian farms.

The costs associated with fatalities and injuries can be broadly broken down into direct and indirect costs. Direct costs may include items such as medical treatment, time off work and workers' compensation claims. In comparison, indirect costs could include damage to equipment, lost productivity, and replacement labour etc. [11, 12] Previous assessments have reported direct costs of fatalities at $160 million/year (AUD 2008) and total costings (direct and indirect) of fatalities, workers' compensation claims and self‐reported injury at $2.35 billion/year (AUD 2012–2013) [12, 13, 14]. More recently (2022), a national report of injury impacts on gross domestic product (GDP) illustrated that if all work‐related injuries and illnesses could be avoided across all industries, Australia's economy would grow by $28.6 billion annually. With specific reference to agriculture, this figure was estimated to be $218 million per year [15]. Even with the most conservative estimate above, it is apparent that there are significant cost implications for Australian agriculture.

Studies focused on the monetary cost of injuries and fatalities help to establish the magnitude of the problem. Furthermore, the significant impacts on health service resources to treat injury, can prompt public and political responsiveness. Given the physical and economic burdens associated with these incidents, having quality data upon which to develop targeted and effective interventions will be crucial to address the highest priority factors that result in injury [16]. This study seeks to describe the pattern and estimated direct economic burdens associated with unintentional deaths and injuries on Australian farms over the past 11 years (2013–2023).

## 2 Method

This is a retrospective descriptive epidemiological study outlining the non‐intentional deaths and workers' compensation injury claims that have occurred on Australian farms. Data for this review have been sourced from two principal sources: (a) the NCIS; and (b) National Dataset for Compensation‐based Statistics (NDS). The population for this data assessment included the Australian and New Zealand Standard Industrial Classification (ANZSIC) codes that are agriculturally based for commodity sectors (4 digit) [17] (Table A1). AgHealth Australia is the only agriculturally focused agency with this access and is subject to strict ethical requirements in terms of data access and use (Justice Human Research Ethics Committee, Department of Justice and Community Safety ‐ Ethics No. CF/19/27527).

### 2.1 Fatalities

The NCIS is the sentinel repository of all deaths reported to a coroner in Australia and New Zealand. Operating since 2000, it provides data from each of the jurisdictions into an electronic database. Following a fatality, a case file is made for each incident by the coroner's court. These are uploaded by the courts to the NCIS, usually within seven days of the incident, with additional details (police narratives of circumstances, autopsy reports, toxicology etc.) added as they become available. Information includes the agent(s) involved in the incident (e.g., objects such as quads and tractors or substance causing the injury). Coronial findings are eventually uploaded once the relevant coroner has assessed the case. When this is done the case is “closed” (which in some instances can take 3–5 years or longer) [18]. AgHealth Australia has access to “open” cases for all but two jurisdictions across Australia and all jurisdictions for “closed” reports to inform its safety recommendations. For this data set of fatalities, the case closure rate was 82% (617/748). In compliance with the ethics agreement and to ensure privacy and confidentiality, coronial data that contain fewer than five cases cannot document precise numbers; however, such cases do add to the respective total of cases. Data in this study are presented from January 2013 through to the conclusion of December 2023 (11 years). All fatalities, including those considered non‐work‐related, were included in the analysis. This method considers that agricultural workers and their families are actively exposed to occupational risks (e.g., tractors, dams) that are necessary to agricultural production. The model, adapted from previous analyses of agricultural death‐related costs, accounts for a comprehensive range of economic impacts [19]. These include the loss of future income, reduced household contributions, insurance payouts, costs associated with WHA and police investigations, premature funeral expenses, coronial services, and emergency medical responses such as ambulance and hospital care. The current model employs the human capital approach developed by Biddle 2004 [20], in line with most occupational and injury studies, as well as injury cost studies in Australia [21]. This approach is considered the most appropriate to estimate the impact of premature death on society [22]. This method includes several limitations, such as the undervaluation of fatalities involving children, women and the elderly, the inability to account for non‐financial externalities (e.g., pain, suffering, grief, friendship, love) and the undervaluation of intellectual, artistic or cultural work to society [23].

Findings were also subject to a sensitivity analysis, with estimates drawing on official Australian Bureau of Statistics Consumer Price Index data [23]. All cost estimates for fatalities were updated to reflect 2023 Australian dollars. There are also well‐documented limitations to this costing process, including the inability of the model to assess the significant social costs associated with fatal events. Hence, it is reasonable to assume that these estimates are conservative and are at a lower level than the total direct and indirect costs. The model's assumptions include estimating annual income for each fatality based on the occupation listed in the coronial records, which was then matched to ANZSCO codes. To determine the projected loss of future earnings, life expectancy data from the Australian Bureau of Statistics (ABS) Life Tables for 2002–2004 was used [24].

The second assumption involved retirement patterns, with individuals assumed to work at 0.9 full‐time equivalent (FTE) until age 71, decreasing to 0.5 FTE by age 75, and considered fully retired (0 FTE) from age 76 onward. The third assumption addressed household production value, which was calculated starting from age 16 and continuing until age 76 for men and age 82 for women [25]. The fourth assumption applied compensation payments in cases where the death was work‐related. The fifth assumption introduced a friction period during the year of death, accounting for 25% of the individual's annual salary.

### 2.2 Workers' Compensation Data

SWA compiles the national data provided by the WHAs in each jurisdiction and has done so since 2000–2001. Data are supplied by SWA in Excel format upon request, and in accordance with requirements for the coronial data, where case numbers are < 5, data are listed as not for publication (np), but do contribute to the total case numbers presented. Data for the period 2013–2014 to 2021–2022 were available (nine years); however, information for the 2021–2022 period was still provisional in nature, as further claims may yet be forthcoming. The information included details on: (a) Agency (what the cause of the injury was); (b) Nature (the type of resulting injury/illness); (c) Mechanism (how the injury/illness occurred); and (d) Bodily location (body part impacted). Information on the economic costs of these workers' compensation claims was only available up to 2020–2021 (eight years). No indexation using the consumer price index was undertaken on the costings associated with these data. The information includes data on the total number of cases, plus the median and total number of work weeks lost, along with the total and median values of workers' compensation paid.

The available workers' compensation data are categorised into two distinct groupings based on time lost as a result of the injury/disease i.e., 0–4 days (minor) or 5+ days (serious). Typically, those injuries requiring 5+ days are more severe and debilitating, plus have a greater impact on the individual and business operations. Additionally, data were categorised into two distinct time periods to allow for some comparison over time from 2013–2014 to 2016–2017 (four years), and from 2017–2018 to 2021–2022 (five years—2022 provisional). To cater for the variation in years assessed, averages were calculated for each period.

## 3 Results

### 3.1 Fatalities

In the 2013–2023 period, there were a total of 748 fatal cases on Australian farms, with 544 (73%) work‐related, 203 (27%) not work related, and one still under investigation. Males dominated the work‐related cases (*n* = 513; 94%), with those 60 years of age and over accounting for almost half of these work cases (*n* = 249; 46%). The agency of the cases involved a broad range, including, but not limited to, the following: tractors (*n* = 118; 16%), quads (*n* = 117; 16%), farm utility (*n* = 52; 7%), side‐by‐side vehicle (SSV) (*n* = 44; 6%), horses (*n* = 29; 4%) and motorbikes (*n* = 29; 4%). These six agents accounted for 52% of all fatal cases (Table 1).

**TABLE 1:** Agents of fatal injury on farms by work‐status (2013–2023).

Agent	Non‐work related	Work‐related	Total	Agent	Non‐work related	Work‐related	Total
Farm vehicles			327	Mobile farm machinery/plant			206
Quad	32	85	117	Tractor	6	112	118
Utility	33	19	52	Forklift	np	+	12
SSV	28	16	44	Bobcat	np	+	8
Motorbike	16	13	29	Bulldozer	np	+	8
Car	+	np	16	Harvesting machine	0	8	8
Helicopter	0	15	15	Slasher/mower/conditioner	0	6	6
Truck	np	+	15	Telehandler	0	6	6
Fixed Wing Aircraft	np	+	12	Other	np	+	40
Trailer	np	+	11				
Gyrocopter	0	6	6	Working Environment			56
Other	+	np	10	Trees (felling)	6	12	18
				Trees (not felling)	np	+	14
Farm Structures			56	Fire/smoke/flame	0	11	11
Dam/Creek/river	+	np	19	Other	np	+	13
Powerlines	np	+	8				
Water tank	np	+	7	Animal			55
Other shed	np	np	5	Horse	16	13	29
Other	np	+	17	Cattle	0	14	14
				Other	np	+	12
Other Classified			47				
Firearms	np	np	6	Unknown		+	+
Other	6	35	41	Total	203	544	747#

*Note:* np, < 5 cases; +, data > 5 cases but not included to retain confidentiality; #, one unknown case.

Abbreviation: SSV, side by side vehicle.

All 748 cases since 2013 were placed into the economic model [1]. As indicated in Table 2, the estimated total direct and indirect impact of fatal incidents since 2013 is almost $1.8 billion (~$164 million per year). While the estimated average costs of a fatality will vary depending on the age and gender of the deceased, the overall average cost of a fatality in 2023 Australian dollars is approximately $2.4 million. Work‐related cases (*n* = 544) totalled $1.24 billion, with an average cost of $2.3 million.

**TABLE 2:** Estimated economic cost of farm‐related fatalities for the 2013–2023 period by age group ($ millions, 2023 dollars).

Age (years)	No. cases	Total ($)^a^	Average ($)^a^
< 15	87	172 540 000	1 985 000
15–24	61	196 070 000	3 215 000
25–34	68	328 350 000	4 830 000
35–44	72	281 800 000	3 915 000
45–54	99	348 470 000	3 520 000
55–64	125	257 455 000	2 060 000
≥ 65	236	194 995 000	825 000
Total	748	1 780 000 000	2 380 000

^a^ All figures rounded.

### 3.2 Workers Compensation Data

As outlined in Table 3, the annual means in both time periods indicate there were more serious claims (5+ days). Although there was a reduction in annual means for minor claims (0–4 days) of approximately 10%, this was offset by a 5% increase in serious claims. There were around 5000 workers' compensation injury claims processed per year.

**TABLE 3:** Workers' compensation injuries annual mean number of claims.^a^

Time lost (days)	Total 2013–2014 to 2016–2017 (annual mean)	Total 2017–2018 to 2021–2022^b^ (annual mean)
0–4 days	9280 (2320)	10 220 (2045)
5^a^ (serious claims)	10 900 (2725)	14 385 (2875)
Total	20 185 (5045)	24 605 (4920)

^a^ Totals may not align exactly as all figures have been rounded.

^b^ Denotes provisional data.

Leading agencies in both periods included animal, human and biological agencies, along with non‐powered handtools, appliances, and equipment (Table 4). There were also a considerable number involving environmental agencies (e.g., hot objects, heat, cold, electricity), mobile plant and transport, plus materials and substances. There was little variation in the agents responsible in the two periods when assessing the annual means.

**TABLE 4:** Agency of all workers' compensation injury claims.^a^

Agency of injury/disease (major group)	Total 2013–2014 to 2016–2017			Total 2017–2018 to 2021–2022^b^		
		Percentage	Annual mean		Percentage	Annual mean
Animal, human and biological agencies	4430	22%	1110	5260	21%	1050
Chemicals and chemical products	335	2%	85	370	2%	75
Environmental agencies	3055	15%	765	4155	17%	830
Machinery and (mainly) fixed plant	1185	6%	295	1490	6%	300
Materials and substances	2575	13%	645	3185	13%	635
Mobile plant and transport	2620	13%	655	3055	12%	610
Non‐powered handtools, appliances and equipment	3799	19%	925	4265	17%	855
Other and unspecified agencies	1695	8%	425	2060	8%	410
Powered equipment, tools and appliances	590	3%	145	765	3%	155
Total	20 185	100%	5045	24 605	100%	4920

^a^ Totals may not align exactly as all figures have been rounded.

^b^ Denotes provisional data.

Traumatic joint incidents followed by wounds and fractures, were highly prevalent (Table A2). These three types of injury accounted for approximately 75% of all claims, with little variation in the means for claims over the two periods.

The majority of incidents (~75%), involved being hit by moving objects, body stressing, along with falls, trips and slips (Table A3). There was little difference in the annual means for the various mechanisms over the two time periods.

As indicated in Table A4, injury to the upper limbs dominated the bodily locations involved and accounted for just under 40% of all cases. In conjunction with injuries to the lower limbs, trunk and head, these four body locations totalled over 90% of cases. There was minimal variation in the distribution of the annual means and their respective totals in the two time periods.

### 3.3 Workers' Compensation Time off and Economic Costs

Table 5 indicates that total time (weeks) off work remained in a range between approximately 55 000 and 70 000 weeks per year. In the period since 2013, the direct total value of workers' compensation claims exceeded $1.5 billion (~$190 million/year). There are also some fluctuations in annual claims costs, with a high in 2017–18 and a reduction since this period. As can be seen in Table 6, there was little change in the median number of weeks lost since 2017–18. Compensation costs associated with serious injury claims (5+ days), comprised virtually all of the costs (~95%) in the period ($1.44 billion).

**TABLE 5:** Workers' compensation injuries all accepted claims (minor and serious) by time and cost^a^ with median values for serious claims (5+ days).

Year	Total time (all accepted)		Total cost (all accepted)	
	Weeks	Percentage	($)	Percentage
2013–14	55 460	11%	167 965 000	11%
2014–15	54 575	11%	160 655 000	11%
2015–16	63 895	13%	222 090 000	15%
2016–17	66 250	13%	215 060 000	14%
Sub Total	240 180	48%	765 770 000	51%
2017–18	72 140	14%	226 480 000	15%
2018–19	65 730	13%	198 585 000	13%
2019–20	66 060	13%	171 384 000	11%
2020–21	58 540	12%	144 145 000	10%
Sub‐Total	262 470	52%	740 595 000	49%
Total	502 650	100%	1 505 000 000	100%

^a^ Totals may not align exactly as all figures have been rounded.

**TABLE 6:** Workers' compensation injuries median values for serious claims (5+ days).

Year	Median time (all accepted—serious claims only)	Median cost (all accepted—serious claims only)
	(weeks)	($)
2013–14	5.4	9310
2014–15	5.2	8950
2015–16	6	11 320
2016–17	6.5	12 250
2017–18	6.9	13 230
2018–19	6.4	12 830
2019–20	6.6	12 660
2020–21	6.8	12 430
Total	6.3	11 750

## 4 Discussion

This is the first assessment of all on‐farm injury related deaths (work and non‐work), in conjunction with a compilation of workers' compensation injury data in Australia. Of the 748 fatalities in the 11‐year period assessed, six agents accounted for over half the cases (52%) –tractors, quads, utes, SSV, horses and motorcycles. Overall, the estimated direct and indirect economic costs of fatalities were in the vicinity of $1.8 billion or $164 million per year. Of these cases, work‐related incidents accounted for 73% (*n* = 544) and 70% of this sum ($1.24 billion). Annually, there were around 5000 workers' compensation injury claims processed per year, costing over $1.5 billion in the eight‐year period (~$190 million per year). As such, the annual economic costs associated with on‐farm injury deaths and workers' compensation injury claims are around $355 million per year.

In concurrence with other studies, fatal work incidents were dominated by males and particularly those over the age of 60 years [8, 26]. These data also reinforce the fact that the majority of on‐farm cases (73%), are related to work. While the remaining 27% of non‐work cases are not insignificant, it does highlight the difficulties in reducing on‐farm fatalities and injuries, as the farm is often also a residence and home, where exposure to some risks is inevitably elevated (e.g., travel distance, isolation and lack of nearby assistance if events occur, etc.) [27, 28]. The importance of targeting key hazards to reduce deaths (and injuries) is also highlighted, with a previous assessment of NCIS data suggesting that known evidence‐based approaches could potentially ameliorate cases for the six leading agents by up to 36% (e.g., rollover protection structures, seatbelts, operator protection devices, helmets) [29].

For the workers' compensation injury data, there has been only marginal change in the overall number or pattern (nature, mechanism and agency) of cases dating back to 2013–2014. Claims involving traumatic joint/ligament and muscle/tendon injury, along with wounds, lacerations, amputations and internal organ damage, were most common. Additionally, fractures and to a lesser extent musculoskeletal and connective tissue diseases were also prevalent. The nature of these injuries is in keeping with the exposure to physically demanding work, machinery/vehicles and animals [30]. Similarly, the mechanisms of injury, with being hit by moving objects being the leading mechanism, also reinforce this pattern. This is also supported by other mechanisms including body stressing, falls, trips and slips. These issues also have a direct parallel with the fatalities data outlined above. From an economic viewpoint, as noted elsewhere [31], the focus should be on reducing serious injury claims (5+ days), given the extensive costs attributable to these cases. While the balance of expenditure on minor claims (1–4 days) is not minimal (~$60 million), this information reinforces the need to prioritise the reduction of severe injury within the sector. Furthermore, targeting such issues is also likely to contribute considerably to reducing minor claims.

In respect to workers' compensation injury data, there are known limitations in terms of the detail available for each specific incident, which allows only broad observations to be made. Data for the current period have also been rounded to further enhance privacy and confidentiality. Furthermore, workers' compensation injury data are unlikely to include information on injuries to owner‐operators and hence under‐represent the true burden of injury in the agricultural sector generally. Additionally, we were unable to align costs directly with the agency, mechanism and nature of workers' compensation claims. However, this is an issue that should be considered in future reviews to more clearly identify the type of claims that could have the largest role in terms of their economic impact.

A strength of this study is that it provides detailed direct estimates of costs for all on‐farm injury deaths and workers' compensation injury claims conjointly, with a conservative annual direct estimate of $355 million/year.

## 5 Limitations

While the deaths data is highly reliable, there may be a small number of cases that could have inadvertently been missed within the NCIS. Although workers' compensation injury claims provide a standardised manner in which to assess injury risk in the sector, there are some known limitations of the data [32]. All employees in Australia, regardless of the industry they work in, must be covered under workers' compensation arrangements. Within Australia, there are over 85 000 farm businesses, of which 99% are family owned and operated [28]. Where these businesses employ staff, they are covered under workers' compensation arrangements. However, unless farm owner‐operators are part of a family trust, they are not covered under such agreements [33]. In these circumstances, while they may have income protection in the event of an injury, these cases are not recorded in the workers' compensation records. In addition, it is known that many farmers (even with workers' compensation in place), may not report cases, for fear of impacting on subsequent coverage fees [32]. Further, cases involving diseases and mental health are known to be under‐reported in the sector [33]. Given these issues, it has previously been estimated that workers' compensation only applies to 58% of all people working on a farm in Australia [34].

Based on these figures, it is feasible that the workers' compensation information under‐represents the true extent of the injury burden within the agricultural industry by around 40%. Consequently, the true economic impact of injury (excluding deaths) could be almost double the approximate $190 million/year identified from these data (i.e., $265 million/year). This would result in an annualised cost of over $430+ million/year if account is also taken of the cost estimates for fatal injuries. Although these costs are significantly lower than have been reported elsewhere ($2.35 billion/year), their study used an alternate methodology and assessed a combination of both direct and indirect costs (which are not included in the workers' compensation data used in this study) [12, 13, 14]. Even at the lower estimate ($430+ million/year), these figures are cause for concern and action.

Both worker health and economic considerations are key components within the sustainability frameworks underpinning agricultural sectors in Australia [3, 4, 5]. Furthermore, terms such as productivity and efficiency are frequently discussed and promoted across the industry [35]. If the direct and indirect costs exceed $430+ million/year as identified in this study, there are significant gains not only in terms of reducing the injury burden but also in relation to the costs associated with these incidents. This articulates well with the focus of sustainability frameworks and should be built upon to reach producers with safety messaging at the farm level. Importantly, the study data provide a baseline of direct costs that can be updated annually for agriculture and potentially individual commodity sectors, as both the fatality (NCIS) and serious injury (workers' compensation) data are finalised. Notwithstanding the limitations noted previously, this information can assist commodity sectors in monitoring their progress in relation to WHS.

There is also additional detail that needs to be assessed to examine variations in work and non‐work cases, particularly in relation to the agencies responsible for injury and the nature of injury. This should also examine in more depth the variations between different age cohorts. Such information would refine the level of detail upon which to develop and focus preventive actions with the farming community.

Future cost–benefit studies are necessary to quantify the economic gains (e.g., potential return on investment) from interventions in key prevention areas, which would support evidence‐based policy and funding decisions. Given the sector‐wide nature of agricultural injuries and fatalities, we acknowledge the need for a coordinated approach to prevention, including integrated safety programmes that span farm types and regions, emphasising the role of government, industry bodies and community organisations. Potential key areas of investment in the short term based on injury prevalence and cost burden include machinery safety upgrades and training, particularly for tractors and quad bikes; mental health support and stress management programmes, which are increasingly linked to injury risk; and targeted education and outreach for young and ageing workers, who represent vulnerable groups. Future research could examine the extent to which institutional systems—such as administrative procedures, bureaucratic structures and regulatory frameworks—facilitate or hinder the reporting of specific claims and compensation cases.

## 6 Conclusion

The comparison between and over time periods in this study illustrates little change in reducing deaths and injuries from incidents. While further gains in health and safety are clearly feasible, these data indicate additional emphasis in addressing risks to persons working within the agricultural industries and living/visiting farms are required. Any gains would not only have benefit for the individuals, their families and the communities involved, but also for the productive outputs of all farm businesses.

## Author Contributions

Carlos Mesa‐Castrillon; data curation, formal analysis, writing – original draft, writing – review and editing. Kerri‐Lynn Peachey: data curation, writing – review and editing, project administration. Tony Lower: conceptualization, methodology, formal analysis, writing – review and editing.

## Ethics Statement

Justice Human Research Ethics Committee, Department of Justice and Community Safety approved the protocol‐ Ethics No. CF/19/27527.

## Conflicts of Interest

The authors declare no conflicts of interest.

## Data Availability

The data that support the findings of this study are available from SafeWork Australia. Restrictions apply to the availability of these data, which were used under licence for this study. Data are available from https://www.safeworkaustralia.gov.au/new‐contact with the permission of SafeWork Australia.

## Appendix A

**TABLE A1:** The Australian and New Zealand Standard Industrial Classification (ANZSIC) codes.

01	Agriculture		
	011	Nursery and floriculture production	
		0111	Nursery production (under cover)
		0112	Nursery production (outdoors)
		0113	Turf growing
		0114	Floriculture production (under cover)
		0115	Floriculture production (outdoors)
	012	Mushroom and vegetable growing	
		0121	Mushroom growing
		0122	Vegetable growing (under cover)
		0123	Vegetable growing (outdoors)
	013	Fruit and tree nut growing	
		0131	Grape growing
		0132	Kiwifruit growing
		0133	Berry fruit growing
		0134	Apple and pear growing
		0135	Stone fruit growing
		0136	Citrus fruit growing
		0137	Olive growing
		0139	Other fruit and tree nut growing
	014	Sheep, beef cattle and grain farming	
		0141	Sheep farming (specialised)
		0142	Beef cattle farming (specialised)
		0143	Beef cattle feedlots (specialised)
		0144	Sheep‐beef cattle farming
		0145	Grain‐sheep or grain‐beef cattle farming
		0146	Rice growing
		0149	Other grain growing
	015	Other Crop Growing	
		0151	Sugar cane growing
		0152	Cotton growing
		0159	Other crop growing n.e.c.
	016	Dairy cattle farming	
		0160	Dairy cattle farming
	017	Poultry farming	
		0171	Poultry farming (meat)
		0172	Poultry farming (eggs)
	018	Deer farming	
		0180	Deer farming
	019	Other livestock farming	
		0191	Horse farming
		0192	Pig farming
		0193	Beekeeping
		0199	Other livestock farming n.e.c.

*Note:* 052, Agriculture, Forestry and Fishing Support Services; 0522, Shearing Services.

**TABLE A2:** Leading workers' compensation injuries claims by major nature of injury (all claims).^a^

Nature of injury/disease (major group)	Total 2013–2014 to 2016–2017			Total 2017–2018 to 2021–2022^b^		
		Percentage	Annual mean		Percentage	Annual mean
Burn	400	2%	100	450	2%	90
Circulatory system diseases	35	0%	10	30	0%	5
Digestive system diseases	205	1%	50	285	1%	55
Fractures	3060	14%	765	4020	16%	805
Infectious and parasitic diseases	155	1%	40	165	1%	35
Intracranial injuries	285	1%	70	450	2%	90
Mental health conditions	195	1%	50	315	1%	65
Musculoskeletal and connective tissue diseases	1550	7%	385	2485	10%	495
Nervous system and sense organ diseases	370	2%	90	660	3%	130
Other claims	90	0%	25	135	1%	25
Other diseases	45	0%	10	25	0%	5
Other injuries	1230	6%	310	1265	5%	255
Respiratory system diseases	45	0%	10	70	0%	15
Skin and subcutaneous tissue diseases	275	1%	70	350	1%	70
Traumatic joint/ligament and muscle/tendon injury	7980	38%	1995	8815	34%	1765
Wounds, lacerations, amputations and internal organ damage	5285	25%	1320	6275	24%	1255
Total	21 195	100%	5300	25 795	100%	5160

^a^ Totals may not align exactly as all figures have been rounded.

^b^ Denotes provisional data.

**TABLE A3:** Leading workers' compensation claims by major mechanism of injury (all claims).^a^

Mechanism of injury/disease (major group)	Total 2013–14 to 2016–17 (annual mean)	Total 2017–18 to 2021‐22^b^ (annual mean)
Being hit by moving objects	6160 (1540)	7740 (1550)
Biological factors	150 (35)	220 (45)
Body stressing	4910 (1225)	5910 (1180)
Chemicals and other substances	520 (130)	550 (110)
Falls, trips and slips of a person	4180 (1045)	5235 (1050)
Heat, electricity and other environmental factors	360 (90)	415 (85)
Hitting objects with a part of the body	2460 (615)	2660 (530)
Mental stress	180 (45)	300 (60)
Sound and pressure	200 (50)	380 (75)
Vehicle incidents and other	1910 (480)	2315 (460)
Total	**21 030 (5255)**	**25 625 (5145)**

^a^ Totals may not align exactly as all figures have been rounded.

^b^ Denotes provisional data.

**TABLE A4:** Workers' compensation claims by bodily location (all claims).^a^

Bodily location of injury/disease (major group)	Total 2013–14 to 2016–17 (Annual Mean)	Total 2017–18 to 2021‐22^b^ (Annual Mean)
Head	2130 (530)	2535 (510)
Lower limbs	5365 (1340)	6775 (1355)
Multiple locations	870 (220)	750 (150)
Neck	330 (85)	455 (90)
Non‐physical locations	190 (50)	315 (65)
Systemic locations	190 (50)	200 (40)
Trunk	3575 (895)	4500 (900)
Unspecified	250 (60)	320 (60)
Upper limbs	8190 (2050)	10 010 (2000)
Total	**21 100 (5275)**	**25 850 (5170)**

^a^ Totals may not align exactly as all figures have been rounded.

^b^ Denotes provisional data.
